# Reference-based genome compression using the longest matched substrings with parallelization consideration

**DOI:** 10.1186/s12859-023-05500-z

**Published:** 2023-09-30

**Authors:** Zhiwen Lu, Lu Guo, Jianhua Chen, Rongshu Wang

**Affiliations:** 1 School of Information, Yunnan University, KunMing, China; 2 Yunnan Physical Science and Sports Professional College, KunMing, China

**Keywords:** Suffix array, CUDA, Genome compression, Reference-based, Parallelization

## Abstract

**Background:**

A large number of researchers have devoted to accelerating the speed of genome sequencing and reducing the cost of genome sequencing for decades, and they have made great strides in both areas, making it easier for researchers to study and analyze genome data. However, how to efficiently store and transmit the vast amount of genome data generated by high-throughput sequencing technologies has become a challenge for data compression researchers. Therefore, the research of genome data compression algorithms to facilitate the efficient representation of genome data has gradually attracted the attention of these researchers. Meanwhile, considering that the current computing devices have multiple cores, how to make full use of the advantages of the computing devices and improve the efficiency of parallel processing is also an important direction for designing genome compression algorithms.

**Results:**

We proposed an algorithm (LMSRGC) based on reference genome sequences, which uses the suffix array (SA) and the longest common prefix (LCP) array to find the longest matched substrings (LMS) for the compression of genome data in FASTA format. The proposed algorithm utilizes the characteristics of SA and the LCP array to select all appropriate LMSs between the genome sequence to be compressed and the reference genome sequence and then utilizes LMSs to compress the target genome sequence. To speed up the operation of the algorithm, we use GPUs to parallelize the construction of SA, while using multiple threads to parallelize the creation of the LCP array and the filtering of LMSs.

**Conclusions:**

Experiment results demonstrate that our algorithm is competitive with the current state-of-the-art algorithms in compression ratio and compression time.

**Supplementary Information:**

The online version contains supplementary material available at 10.1186/s12859-023-05500-z.

## Introduction

Genome sequencing technology is still moving towards high speed and low cost and has made significant breakthroughs. Such breakthroughs have attracted a large number of scholars to participate in the research of biological genome data, including how to efficiently transmit and store a large amount of genome data generated by high-throughput sequencing technology. The ERGC compression algorithm was proposed in [1], which separates the target sequence and the reference sequence into segments of a fixed length, creates a hash table for each segment, and then performs matching searches and extends these matches. After the process of searching and extending matches, the algorithm processes the matching results and then compresses the temporary files by the PPMD algorithm. It is worth noting that the compression performance of ERGC is excellent in compressing the target sequence that has a good similarity with the reference sequence, and reduces the memory usage of the algorithm. However, it performs worse in compressing the target sequence that is not so similar to the reference sequence. Experimental results show that the compression performance of ERGC is worse than that of the previous algorithms [2, 3] on some genome datasets [4]. In 2017, the HiRGC compression algorithm was proposed in [5]. In this algorithm, all letters except for {A.C.G.T} are deleted from the input target and reference sequences during the pre-process, and lowercase letters are converted to uppercase letters. Then, a hash table for the pre-processed reference sequence is constructed for searching of the longest matches, which solves the problem of separating a long match into two or more short matches in ERGC, thus obtaining better matching results and further improving the compression ratio of the algorithm. It also improves the speed of establishing the hash table. However, HiRGC requires much more memorys for the hash table than ERGC does. Since HiRGC uses the longest matching strategy when searching for matches, resulting in a large variation in the distance between the positions of two neighboring matches, which in turn deteriorates the final compression result. The SCCG compression algorithm was proposed in [6], which dynamically combines the advantages of the matching methods of ERGC and HiRGC, and carefully considers the effect of the length and the position of the matched sub-strings on the compression ratio. The target and the reference sequences are pre-processed and then the local matching strategy is adopted first, but, if this strategy fails, the global matching strategy is adopted. The local matching strategy segments the target and the reference sequences into fixed length substrings, to improve the efficiency of the following incremental coding. The global matching strategy expands the search scope and improves search efficiency. In 2019, the ECC algorithm was proposed in [7], which efficiently selects a good reference sequence in a candidate set for the above compression algorithms to obtain better compression results. The HRCM compression algorithm was proposed in [8], which utilizes the second-order matching method in GDC-2. At the same time, it proposed a matching algorithm for lower case letters and no longer records the information of lower case letters in the target genome sequence. The algorithm achieves good compression results. All algorithms mentioned above use a greedy strategy to search, in the reference sequence, for the longest matching string prefixed with the current *kmer* in the target sequence, while ignoring LMS (also known as Maximum exact matches (MEM)) between the target and the reference genome sequences. Scholars have been doing much research on MEM between different sequences. The essaMEM algorithm was proposed in [9], the algorithm improves over the SSA [10] to find the MEM between two sequences. In 2014, the kmacs algorithm was proposed in [11], which uses SA to establish the LCP array of adjacent suffix and record the length of LCP for the searching of MEMs between different sequences. The copMEM [12] algorithm was proposed in 2018, which search MEMS by using coprime sampling technology that based on bfMEM [13], an algorithm that reduces the higher memory requirement in establishing a hash table and increases the speed of searching MEM by keeping kmers that do not collide in the hash table through a bloom filter. An algorithm was proposed in [14], which use the longest common subsequence shared between the reference and the target sequences for the compression of the target sequence. The memRGC [15] utilizes MEMs between the target and the reference sequences to compress the target sequence. The key idea of the algorithm is to repeatedly detect the maximum exact matches between the target and the reference sequences by combining bfMEM and copMEM methods, and extend these matches in both directions. The temporary file storing the matching results is compressed by BSC, and the algorithm achieves a satisfactory compression ratio, but the algorithm does not consider the reverse complementary of the to be compressed genome sequence. Although memRGC provides a multi-thread parallel mode, it still compresses one sequence per thread and does not involve multi-thread parallelization acceleration of the compression of a sequence. SparkGC [16] is based on Spark and utilizes multiple nodes to compress large collections of genomes. This algorithm contains two steps: the first-order and the second-order compression. MEMs between the target and the reference sequences are searched and encoded as tuples during the first-order compression. These tuples will be processed by the method in GDC-2 during the second-order compression to improve the compression ratio.

On the other hand, none of the researchers mentioned above have carefully considered the parallelization of their algorithms for multi-core CPUs when compressing a single sequence. On the basis of this consideration, we propose an algorithm which compresses a single genome sequence by searching LMSs between the target and the reference genome sequences. Meanwhile, the proposed algorithm uses GPUs and multi-core CPUs to create the searching structure based on SA and the LCP array in parallel. According to the characteristics of SA and the LCP array, we use multi-threads programming to complete the selection of appropriate LMSs in parallel. Finally, the selected LMSs are encoded to generate a temporary file to store the matching results, and the file is compressed. Taking into account the biological property of the reverse complementary in genome sequences, we also constructed reverse complementary sequences for the reference genome sequence to increase the length of the matched LMSs. Experiment results show that our algorithm is competitive with the current state-of-the-art algorithms in terms of the compression result and the compression time.

## Materials and methods

**Fig. 1:**
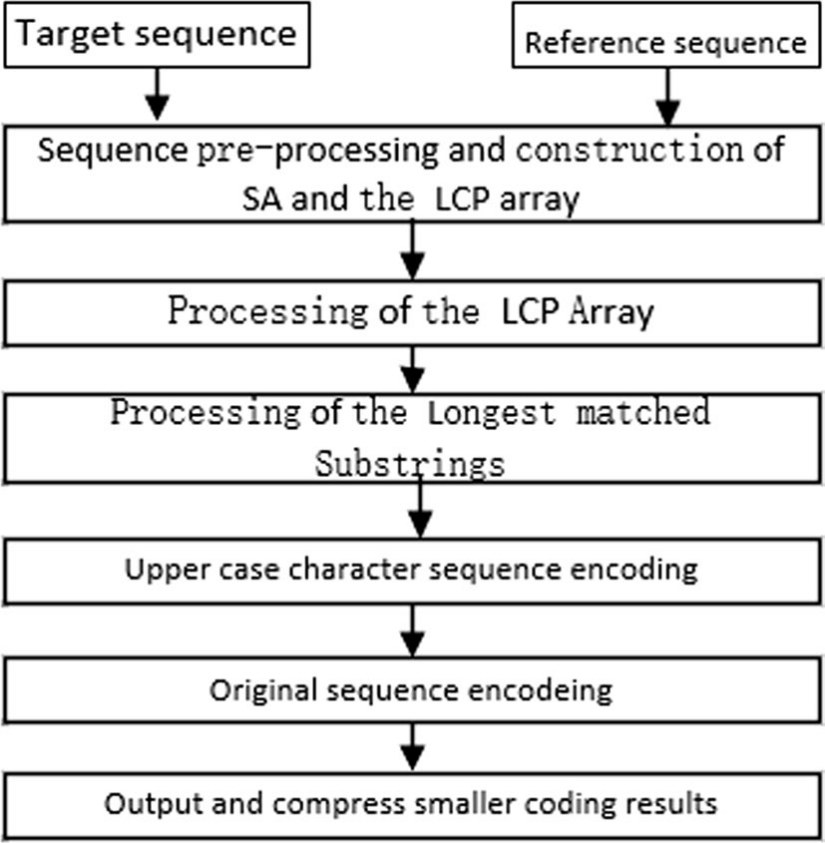
Schematic diagram of proposed algorithm

The input target and reference genome sequences are pre-processed first, the reverse complementary sequence of the input reference genome sequence is constructed, then SA and the LCP array are constructed based on the pre-processed sequences as the index structure for searching LMSs, the target genome sequences are compressed by iteratively finding the remaining LMS using the index structure and encoding the corresponding information. The specific workflow of the algorithm is shown in Fig. 1.

### Sequence pre-processing and construction of SA and the LCP array

During the sequence pre-processing, all lowercase letters in the target and reference genome sequences are converted into uppercase letters, all letters except for {A, C, G, T} in the reference sequence are deleted, the reverse complementary sequence of the reference sequence are constructed and concatenated with the original sequence to form a longer reference sequence. In this way, the lengths of matched LMSs can be increased. When converting lowercase letters into uppercase letters in the target sequence, the position and length information of lowercase letters in the target sequence should be recorded and eventually compressed for decoding. The target and the reference sequences are concatenated, with a $ symbol between them, to form the input sequence $${\text{S}} = {\text{S}}[0]$$$$....{\text{S}}[{\text{n}}]$$ for the construction of SA, where $$n$$ is the total length of the input sequence. Assume that the target sequence is ‘CCCTAG’, the reference sequence is 'ACCTCT', the reverse complementary sequence of the reference sequence is ‘AGAGGT’, and the input sequence for the construction of SA is then ‘CCCTAG$ACCTCTCTAGAGGT’. An example of SA and the LCP array constructed based on the input sequence ‘CCCTAG$ACCTCTAGAGGT’ is shown in Table 1: the suffix array is the index of all suffixes of the input sequence sorted by dictionary order, that is, $$SA\left[ i \right] = m$$ means that the $$i$$th suffix of all suffixes in the input sequence sorted by dictionary order is the suffix starting from the $$m$$th letter of the input sequence. The LCP array represents the length of the longest common prefix between the current suffix and the previous suffix, that is, $$LCP\left[ i \right] = x$$ means that the two suffixes represented by $$SA\left[ i \right]$$ and $$SA\left[ {i - 1} \right]$$ have at most $$x$$ identical prefix letters from the first letter.

**Table 1:** Suffix-array and LCP array

Input: CCCTAG$ACCTCTAGAGGT			
*i*	*SA[i]*	*Suffix*	*LCP[i]*
0	6	$ACCTCTAGAGGT	–
1	7	ACCTCTAGAGGT	0
2	4	AG$ACCTCTAGAGGT	1
3	13	AGAGGT	2
4	15	AGGT	2
5	0	CCCTAG$ACCTCTAGAGGT	0
6	1	CCTAG$ACCTCTAGAGGT	2
7	8	CCTCTAGAGGT	3
8	2	CTAG$ACCTCTAGAGGT	1
9	11	CTAGAGGT	4
10	9	CTCTAGAGGT	2
11	5	G$ACCTCTAGAGGT	0
12	14	GAGGT	1
13	16	GGT	1
14	17	GT	1
15	18	T	0
16	3	TAG$ACCTCTAGAGGT	1
17	12	TAGAGGT	3
18	10	TCTAGAGGT	1

As the creation of the suffix array requires all suffixes of the input sequence to be sorted in alphabetical order, this process is time-consuming and the time spent in the suffix array construction would account for the largest proportion of the running time of the entire algorithm. In recent years, as SA has been used in many studies, fast construction methods for SA have been proposed. The pDC3 [17] algorithm is the parallelization implementation of the DC3 suffix array construction algorithm [18] on distributed computers. Since a large number of CPU cores are required to obtain the desired performance when building the suffix array using multi-core CPUs, but CPU cores are more frequently occupied by users in multi-user systems, CPU core-based parallel SA construction methods are mostly implemented on clusters of computers to make full use of the free cores on each machine. In contrast, GPU is typically much less occupied than CPU on a normal computer. An algorithm [19] use GPU to achieve the parallel acceleration of the DC3 algorithm. A better implementation of the parallel construction of the suffix array using GPUs was proposed in [20], therefore it is employed to accelerate the construction of the suffix array in our work. This algorithm segments the input sequence based on the number of GPUs and assigns the segmented sequences to all GPUs. Each GPU uses the prefix doubling technique to sort all suffixes in the assigned sequence, and records the sorting result and the starting positions of the suffixes in the whole sequence in a global array. Finally, the algorithm complete the construction of the suffix array by sorting the global array. Experimental results show that the algorithm greatly speeds up the construction of the suffix array.

To our best knowledge, there is no good algorithm for the construction of the LCP array specifically using GPU features at present. The running time improvement will be limited if merely implementing the existing LCP array construction algorithm in GPU. Even if a GPU algorithm were specifically designed for LCP array construction, the running time improvement would not has great impact on the overall algorithm execution efficiency, since we can use our multi-thread LCP construction algorithm based on a linear LCP construction strategy in [21] to build an LCP array for a 750 Mb sequence in less than 10 s using 20 CPU cores. Therefore, multi-thread programming is applied to construct the LCP array using CPU cores. After creating the suffix array, we segment the suffix array according to the available number of CPU cores and assign the construction of the LCP array of each suffix array segment to a CPU core. During the process of the segmentation, we select the last suffix of the previous segment as the first suffix of the next segment to calculate the length of the LCP between adjacent segments. After the segmentation, we calculate the LCP array for each segment by using the algorithm in [21]. Finally, the LCP array for each segment is concatenated to construct the global LCP array.

### Processing of the LCP array

If the adjacent suffixes are both from the target or the reference sequence, the corresponding value in the LCP array is useless for searching LMS of the two sequences. We should filter out those elements in the LCP array corresponding to adjacent suffixes both from the reference sequence. Based on the comparison of the values in SA with the length of the target sequence, it can be determined that a suffix comes from the reference sequence when its corresponding value in SA is greater than the length of the target sequence. For example, the values in $SA\left[ {12} \right]$*,* $SA\left[ {13} \right]$ in Table 1 are not less than the length of the target sequence, which is $6$, it means that the two suffixes come from the reference sequence. The value in $SA\left[ 8 \right]$ is less than $6$, while the value in $SA\left[ 9 \right]$ not less than $6$, meaning that the two suffixes are from the target and the reference sequences respectively. This filtering process aims to filter out all lcp array elements whose corresponding suffixes are both from the reference sequence only. In this process, if the length of the target sequence is *tarlength*, SA is traversed one by one first to find a suffix that comes from the target sequence, i.e. $SA\left[ i \right] < tarlength$. Then, find in both directions for the nearest adjacent suffixes both from the reference sequence, whose indexes in SA are represented by *rp1* and *rp2.* i.e. $SA\left[ {rp1} \right] < tarlength$ and $SA\left[ {rp2} \right] < tarlength$ but $rp1 < i$ and $rp2 > i$ by using the following equation,

1 $$\begin{array}{*{20}c} {plcp\_len = \mathop {\min }\limits_{rp1 < p \le i} \left( {LCP\left[ p \right]} \right) } \\ {nlcp\_len = \mathop {\min }\limits_{i < n \le rp1} \left( {LCP\left[ n \right]} \right) } \\ {lcs\_len = \max \left( {plcp\_len,nlcp\_len} \right)} \\ \end{array}$$

we can find the longest common substring with length $lcs\_len$ in the reference sequence for the substring in the target sequence, which is the $i$th suffix in SA.

While filtering the LCP array, we create an array of structure with size equal to the length of the target sequence, in which $struct\left[ i \right]$ records 3 parameters: the starting position of an LMS in the target sequence, the starting position of this LMS in the reference sequence, and the length of this LMS. Its index value *i* corresponds to the starting position of this LMS in the target sequence. The details of the selection process can be described with the pseudo-code in Algorithm 1.

With the example in Table 1, when $i = 6$*, *$SA\left[ i \right] = 1 < 6$*,* that is, the starting position of the LMS in the target sequence is $1$. W*e* determine $rp1$ first. Since we need $rp1 < i$*,* if $rp1 = 5$, then ${ }SA\left[ {rp1} \right] = 0 < 6$, it means that the ${\text{rp}}1$-th suffix comes from the target sequence. When $rp1 = 4$, $SA\left[ {rp1} \right] = 15 > 6$, it means that the $rp1$-th suffix comes from the reference sequence. Therefore,${\text{ rp}}1 = 4$. Since ${ }LCP\left[ 5 \right] = 0 < LCP\left[ 6 \right] = 2$, $p = 5$ and the starting position of the suffix in the reference sequence is ${\text{SA}}\left[ {{\text{rp}}1} \right] - {\text{tarlength }} = { }15 - 6 = 9$, ${\text{plcp}}\_{\text{len}} = {\text{LCP}}\left[ {\text{p}} \right]{ } = 0$. Next, ${\text{ rp}}2$ is determined. Since we need ${\text{ rp}}2{ } > {\text{ i}}$, if ${\text{ rp}}2{ } = 7$, then ${\text{ SA}}\left[ {{\text{rp}}2} \right]{ } = { }8 > 6$, it means that the ${\text{ rp}}2$-th suffix comes from the reference sequence,${\text{n}} = 7$. The starting position of this suffix in the reference sequence is $SA\left[ {rp2} \right] - tarlength = 8 - 6 = 2$*.* $nlcp\_len = LCP\left[ n \right] = 3$; *Since* $plcp\_len = 0 < nlcp\_len = 3$, $lcs\_len = 3$ and the start position of the LMS in the reference sequence is $2$; That is, the starting position of the LMS in the target sequence is 1 and its starting position in the reference sequence is 2, the length of the LMS is 3. Based on the matched result, the components of ${ }struct\left[ 1 \right]$ is determined, they are $struct\left[ 1 \right].tar = 1$*,* $struct\left[ 1 \right].ref = 2$*,* $struct\left[ 1 \right].lcs\_len = 3$.

When we use multi-thread programming to complete the selection of appropriate LMSs in parallel using multiple CPU cores, the LCP array cannot be simply segmented to each thread equally according to the number of threads. Since we need to compare the lengths of two LCPs, the length of the previous LCP and that of the next LCP of the current suffix whose starting position has to be in the target sequence, to obtain the LMS, we need to ensure that the starting positions of the first and the last suffixes of each suffix array segment come from the reference sequence. For the first suffix of the segment, we need to determine whether its starting position comes from the reference sequence. If it comes from the target sequence, we will iteratively check the starting position of each previous suffix until we find the one whose starting position comes from the reference sequence. For the last suffix of the segment, if its starting position in the target sequence, we will check each next suffix until a suffix with its starting position in the reference sequence is found. The specific running time of this process is described in details in the experiment results.
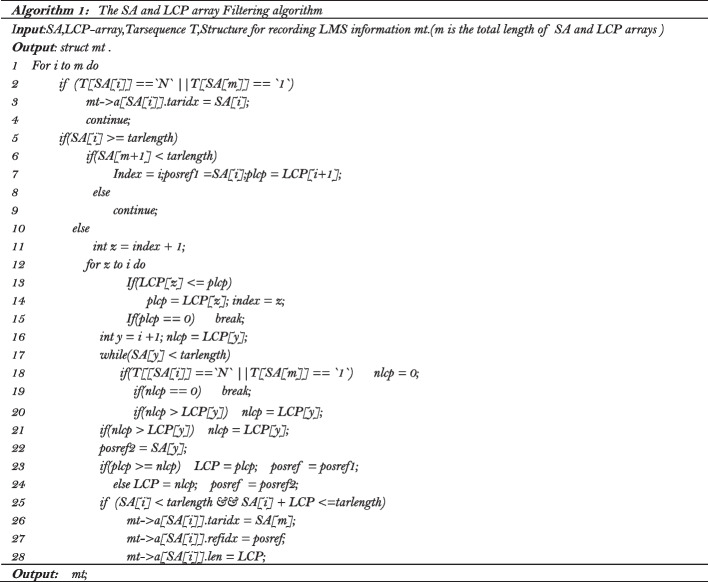


### Processing of the longest matched substrings

After completing the processing of the LCP array, we obtain an array of the structure ${ }S[]$ with its size equal to the length of the target sequence. The element *i* in the array records the information of the substring starting from position *i* of the target sequence, its corresponding LMS in the reference sequence and length of this LMS. However, these LMSs are not necessarily all valid LMSs. There are large number of cases where a shorter LMS are contained in a longer *LMS* and LMSs overlap with each other. Therefore, $S[]$ should be traversed to filter out those LMSs which are contained in other LMSs and LMSs overlapping with each other should be processed.

**Fig. 2:**
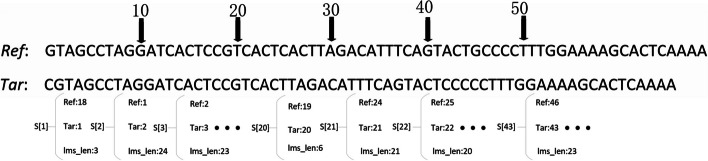
Example of overlap and contain of LMS

Since the index value of $S[]$ corresponds to the start position of the LMS in the target sequence, we traverse $S[]$ according to the index value from small to large first. All LMSs contained in other LMSs will be deleted, and LMSs with length less than the predetermined kmerlength will also be discarded. LMSs overlap with each other will be retained at this time. With the example in Fig. 2, the target sequence is *Tar* and the reference sequence is $Ref$*,* after the processing of the LCP array, we can obtain the structure $S[]$*.* For example $S\left[ 2 \right]$*,* where the value of its component $S\left[ 2 \right]$*. tar* indicates the starting position of an LMS in the target sequence is 2, and the value of its component $S\left[ 2 \right]$*.ref* indicates the starting position of this LMS in the reference sequence is 1, and the value of $S\left[ 2 \right].lms\_len$ indicates the length of this LMS is 24. It can be seen that the LMS with starting position from 3 to 20 in the target sequence are contained in the LMS with starting position of 2 in the target sequence. That is, $S\left[ {2\left] {.tar + S} \right[2} \right].lms\_len > S\left[ {3\left] {.tar + S} \right[3} \right].lms\_len$,…, $S\left[ {2\left] {.tar + S} \right[2} \right].lms\_len > S\left[ {20\left] {.tar + S} \right[20} \right].lms\_len$*.* Therefore, $S\left[ 3 \right]$ *to* $S\left[ {20} \right]$ are deleted but $S\left[ 2 \right]$ is retained*.* The LMS with the start position of 2 in the target sequence overlaps with the LMS with the start position of 21 in the target sequence. That is, $S\left[ {2\left] {.tar + S} \right[2} \right].LMS\_len < S\left[ {21\left] {.tar + S} \right[21} \right].LMS\_len$*.* Therefore, $S\left[ {21} \right]$ will be retained too.

After all those LMSs contained in longer LMSs are deleted, $S[]$ only contains independent LMSs and LMSs overlap with each other. Since the length of LMS greatly affects the compression result, that is, the longer LMS, the smaller the final compression result. Therefore, we sort $S[]$ according to the lengths of the LMSs from long to short and then traverse the sorted array $S[]$, then the longest of the LMSs is recorded. If an LMS overlaps with an already recorded LMS, then the substring of the LMS is truncated, only the part of the substring not covered by the already recorded LMSs is retained and a new LMS is formed. If the length of the new LMS is less than *kmerlength*, it is discarded. Otherwise, all LMSs in ${\text{S}}[]$ not recorded yet are sorted again according to their lengths. The above procedure is repeated until all LMSs are recorded. With the example in Fig. 2, after those LMSs contained in longer LMSs are deleted, $S\left[ 2 \right]$, $S\left[ {21} \right]$, $S\left[ {43} \right]$ are retained, we sort $S[]$ according to the length of LMSs in it. $S\left[ 2 \right]$ is recorded first, and then $S\left[ {43} \right]$ is recorded too. The first 6 characters of the LMS in $S\left[ {21} \right]$ overlaps with that in $S\left[ 2 \right]$*,* the LMS in $S\left[ {21} \right]$ is then truncated and to form a new LMS which can be represented by the LMS in $S\left[ {27} \right]$*.* Since $S\left[ {27} \right].lms\_len = 15 < kmerlength$, this LMS is discarded. The details of the filtering process of the longest matched substrings can be described with the pseudo-code in Algorithm 2.

The LMSs to be processed in the above procedure are not independent of each other and some short LMSs are created after the processing of longer LMSs. The parallel processing of $S[]$ is not feasible by simply segmenting $S[]$ into blocks, therefore we do not consider the parallelization of this step.
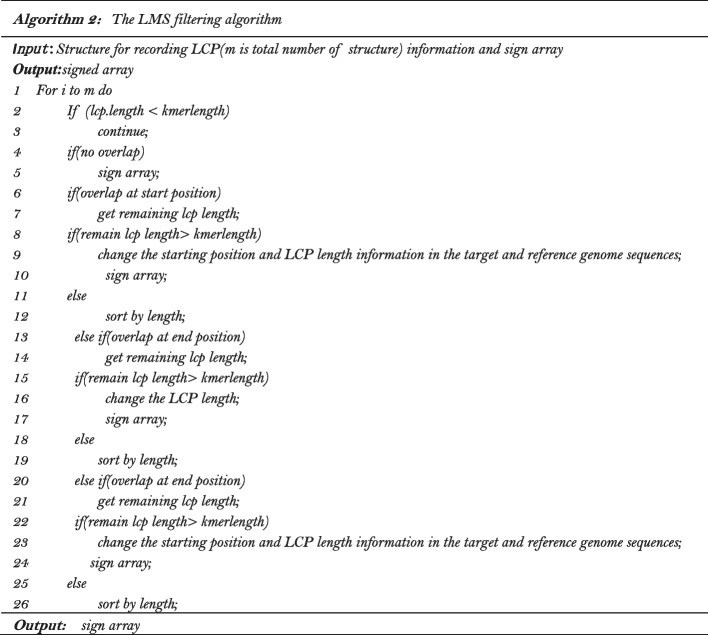


### Encode

Valid LMSs are recorded by the previous procedures and are used to encode the target sequence from the beginning to the end. A substring in the target sequence for which an LMS in the reference sequence can be found is encoded by the position of the LMS in the reference sequence and the length of this LMS. Encoding results and the mismatch letters (substrings for which no LMS can be found in the reference) are stored in a temporary file. For the first encoded LMS, its position in the reference sequence and its length are recorded in the temporary file. For the following encoded LMSs their positions are encoded by the delta coding.

Obviously, it is not that longer LMSs always result in better compression performance. If the position of the current LMS is too far from the location of the last encoded LMS, the results of subsequent delta coding will be worse, resulting in poor overall compression performance. Therefore, when processing the current LMS, it is necessary to consider the distance between its position and the position of the last encoded LMS. If the distance is short, the current LMS is directly used for coding. If the distance exceeds a specific threshold, we will try to start from the end position of the previous LMS in the reference sequence and find the possible substring that can match the current substring letter by letter in the reference sequence. If the length of the letter by letter matching substring exceeds 90% of the length of the existing LMS, we will give up encoding the LMS and encode the result of the letter by letter matching. Otherwise, the LMS is still used for encoding. It is worth noting that when two adjacent LMSs in the target sequence exactly correspond to two adjacent LMSs in the reference sequence, it is obvious that the letters between the adjacent LMSs and the corresponding LMSs in the reference sequence do not match. If the number of mismatched letters between the target sequence and the reference sequence is equal, instead of encoding the positions of these two LMSs separately we only encode the position of the first LMS in the target sequence to further improve the compression performance. As shown in Fig. 2, $S\left[ 2 \right]$ and $S\left[ {43} \right]$ correspond to adjacent LMSs both in the target sequence and the reference sequence, the mismatched string in the target sequence between $S\left[ 2 \right]$ and $S\left[ {43} \right]$ is 'atccctaag', and $S\left[ {43} \right].Ref - \left( {S\left[ {2\left] {.Ref + S} \right[2} \right].lms\_len} \right)$ equals to the number of letters in the string 'atccctaag'. The encoding result should be ' 2,23atccctaag, 23' instead of '2,23atccctaag9,23', the position of the next LMS is omitted.

As the position offsets of LMSs depend on the positions of all LMSs when encoding the recorded LMSs using the delta coding, we do not consider the parallel processing of this procedure to ensure the correctness of the encoding results. During the final compression, the BSC (http://libbsc.com) compressor is used in this work.

## Result

We report the compressed file sizes, compression time and memory consumption of the proposed algorithm in this section. All experiments were implemented in a Red Hat Linux 7.9 (64-bit) server with 2 RTX6000 GPUs with 24GB of RAM, and 2 * 2.6 GHz Intel Xeon Gold 6240 CPUs (18 cores) with 256GB RAM.

For the comparison of the compression performance, an important algorithm to be addressed is SparkGC. It has excellent performance for the compression of large collections of genomes [16], but it performs weak for the compression of small number of genomes. Since in this algorithm, the information about previously compressed chromosomes is also used for the compression of later chromosomes. During decoding, it is necessary to firstly decode the previous chromosomes in order to successfully decode the current chromosome. In practical applications, if the user only needs the last chromosome, this algorithm needs to decode all previously compressed chromosomes before decoding the last chromosome. This characteristic also weakens the efficiency of genome data transmission. For comparison, we have listed the compression results of the proposed algorithm and that of the HiRGC, SCCG, memRGC, SparkGC. For the experiment, we select the genomes that have been used by all the above algorithms as the test data sets which are shown in Table 2.

**Table 2:** Test data

hg17	ftp://hgdownload.soe.ucsc.edu/goldenPath/hg17/chromosomes/
hg18	ftp://hgdownload.soe.ucsc.edu/goldenPath/hg18/chromosomes/
hg19	ftp://hgdownload.soe.ucsc.edu/goldenPath/hg19/chromosomes/
hg38	ftp://hgdownload.soe.ucsc.edu/goldenPath/hg38/chromosomes/
KO131	ftp://ftp.kobic.re.kr/pub/KOBIC-KoreanGenome/KOREF_20090131/fasta/
KO224	ftp://ftp.kobic.re.kr/pub/KOBIC-KoreanGenome/KOREF_20090224/fasta/
HuRef	https://www.ncbi.nlm.nih.gov/nuccore Accession: CM000462-CM000485
YH	ftp://climb.genomics.cn/pub/10.5524/100001_101000/100015/fa/

### Compression performance

**Fig. 3:**
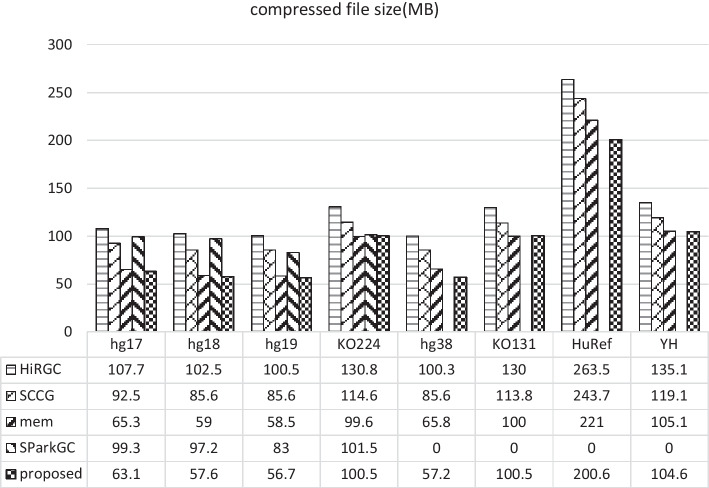
Compression results for 8 genomes

In these 8 genomes, each genome is used in turn as the reference genome to compress the other 7 genomes, each with a raw size of approximately 3 GB. SparkGC is based on Apache Spark, which is run on a 5-node cluster and each node has 20 CPU cores. However, since we do not have such environment, we execute the SparkGC algorithm on a single server. SparkGC generates one intermediate file for each chromosome of a genome, and then chromosomes with the same number in all genomes are compressed into the same final file using BSC. In our experiment, we call the BSC compressor by command line to compress these intermediate files to obtain 24 final compressed files for all genomes. For other algorithms, the intermediate files for 7 genomes are compressed into 7 final files. By adding the sizes of the final files produced by each algorithm the compression results are obtained (Fig. 3).

From Fig. 3, it can be seen that out of the 8 groups, the proposed algorithm is the best in 6 groups, and the compression results of the remaining 2 groups are almost the same as those of the best algorithm. Due to the fact that we execute the SparkGC algorithm on a single server, which is different from its original running environment, some problems occur when using hg38, KO131, HuRef, and YH as references, the intermediate files can not be generated. Therefore, we cannot obtain the final compression results, which are represented by 0 in Fig. 3.

Meanwhile, we also report the detailed compression results for each genome. During the experiment, each genome serves in turn as the reference for the compression of the remaining 7 genomes and 7 compressed files are generated, which are obtained by using BSC compressor to compress the intermediate files for 24 chromosomes. The compression results for the 56 pairs of genomes are presented in Additional file 1: Table S1. However, SparkGC is not compared here, since SparkGC compresses the same chromosome of all genomes into a final file, it cannot generate the corresponding compression result for each genome, which is different from other compared algorithms. Among the 56 genome pairs, the proposed algorithm performs the best in 40 pairs. Although we have 16 pairs of compression results that are worse than memRGC, but most of the differences are within 3%, and the overall compression ratio of the proposed algorithm for all 56 genomes is 4.3% better than that of memRGC.

### Compression time

**Fig. 4:**
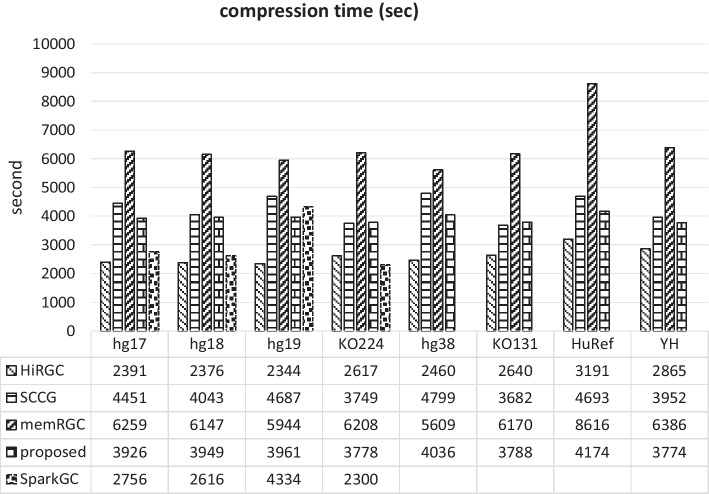
Compression time result for 8 genomes

The compression time results corresponding to the compression of genomes in Fig. 3 are presented in Fig. 4. It should be noted that it is unfair to compare the running time of SparkGC on a single server, since the parallelization advantage of SparkGC on large collection of genomes can not be presented. Anyway, its running time results in our environment are also provided. From Fig. 4, it can be seen that HiRGC is the fastest algorithm. However, the compression results of the proposed algorithm are obtained using 2 GPUs in our system, if more GPUs can be used, better results will be obtained. Considering that the proposed algorithm outperforms HiRGC in compression results by about 30%, and we believe that the current running time results are still worthwhile. The detailed compression time results for all 56 pairs of genomes are shown in Additional file 1: Table S2. Since SparkGC cannot generate the compression result for each genome, its compression time results are not included in this table.

Although the memRGC algorithm provides a multi-thread mode, but one sequence has to be compressed using one thread. i.e. when compressing 24 chromosomes of one genome, memRGC can only utilize a maximum of 24 threads and cannot improve the compression time further by using multiple threads for the compression of a single chromosome sequence. However, our algorithm can accelerate the compression of one chromosome by using multiple threads. The left two columns in Table 3 show the running time of the two algorithms when compressing genomes chromosome by chromosome. It can be seen from Additional file 1: Table S2 that the compression time varies quite significant when memRGC compresses the same genome by using different reference genomes. For example, the compression time of memRGC for hg17-hg18 pair is 1221 s, while the compression time for hg38-hg18 is 566 s. More than that, the compression time of memRGC on different data sets also varies quite significant. On the contrary, our algorithm has a relatively stable running time between 500 and 650 s regardless of which data set is compressed.

**Table 3:** Running time of different thread numbers

Time (s)	Number of threads	30	20	10	1
KO131-KO224 (chr1.fa)	Build LCP	6.3	6.43	10.48	82.44
	Filter SA&LCP	1.86	2.25	4.05	31.38

For hg38-hg17 pair, we have found 9202 LMSs and their average length is 26,679, but for HuRef-hg38 pair, we have found 361,517 LMSs and their average length is 672. It can be seen that our algorithm is insensitive to the number of LMSs that have been found.

### Execution time under different number of threads

In addition, we also analyzed the impact of the number of threads on the running time of the parallel execution parts of our algorithm. For example, we compress the first chromosome of KO131-KO224 pair, the running time of the parallel execution parts of our algorithm in different number of threads are shown in Table 3. It can be seen that the running time speeds up as the number of threads increases from 1 to 30. However, as can be seen in Table 3, when the number of threads is increased from 20 to 30, the improvement of the running time is not so obvious. Since the experiment platform of our algorithm has only 36 CPU cores. Since some CPU cores have to be occupied by the operating system and other users, there are not enough CPU cores for the execution of all threads when the number of threads is set more than 20.

### Memory usage

In terms of memory usage, each element in the target and the reference sequence and reverse complementary sequence of reference sequence occupies 1 byte, and each element in the SA and LCP arrays occupies 4 bytes. There are 3 elements in the structure $S - array$ and each element occupies 4 bytes. If the target and the reference sequences are both 250 MB, the proposed algorithm requires less than 15GB, which can be observed by the “top” command in the Linux system. For the compared algorithms, HiRGC requires less than 8 GB, SCCG requires less than 8 GB, memRGC requires less than 2 GB on the single thread mode, SparkGC requires less than 7 GB.

### Time and memory usage of decompression

**Fig. 5:**
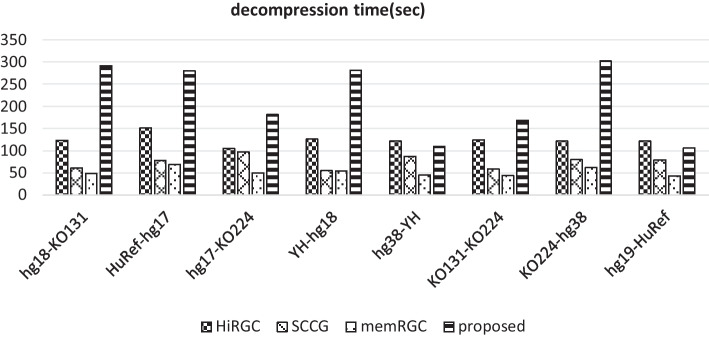
Decompression time of each algorithm

In terms of decompression time, we randomly select several pairs of genomes for experiment, the results are presented in Fig. 5. In this figure, the decompression time for hg18-KO131 means that KO131 is the target genome with hg18 as the reference. Since the proposed algorithm utilizes the reverse complementary sequence of the reference sequence, it is also necessary to construct the reverse complementary sequence of the reference sequence during decompression. Therefore, its performance on decompression time is not so good.

For the memory usage of decompression, all algorithms require less than 1 GB during the decompressing of the longest sequence.

## Conclusions

The proposed compression algorithm uses the suffix array and the longest common prefix array to search the longest matched substrings between the target and the reference sequences for the compression of genome data. The key of the algorithm lies in repeated filtering of the suffix array (SA) and the longest common prefix array(LCP) to obtain longest matched substrings. During filtering SA and LCP, the similarity between the target and reference sequences does not affect the speed of the proposed algorithm, since the algorithm completes filtering by traversing the entire sequence. Therefore, this algorithm has a relatively stable compression time when compressing different genomes. In addition, the parallelization consideration of the algorithm accelerates the compression time. Experiment results demonstrate that the proposed algorithm is also competitive with the state-of-the-art algorithms in terms of the compression ratio and the compression time.

### Supplementary Information

**Additional file 1.** Details of 56 genomes experimental results.

## Data Availability

The C++ code of our algorithm is available at https://github.com/zhi-wen-Lu/LMSRGC. Operating systems: Linux. Programming language: C++. Other requirements: GCC 5.4.0/Cmake 3.8 or greater/CUDA on the path.
