# Supplementary material for: Reference-based genome compression using the longest matched substrings with parallelization consideration
Source: BMC Bioinformatics. 2023 Sep 30;24:369. doi: 10.1186/s12859-023-05500-z (PMC10544193; doi:10.1186/s12859-023-05500-z)
Supplement: Supplementary file 1 — Additional file 1. Details of 56 genomes experimental results. [file 12859_2023_5500_MOESM1_ESM.docx]

**Additional file 1**

The compression results for each pair genome are presented in Table 1, the file sizes are in megabytes (MB), for each pair of target and reference genome sequences the best compression result is highlighted in bold, the compression gain is calculated by formula (1).

$$\frac{\text{compressed size of other algorithm-compressed size of ours}}{\text{compressed size of other algorithm}}\text{*}100\text{\% (1)}$$

|  | Compressed file size(mb) | | | | | | | |
| --- | --- | --- | --- | --- | --- | --- | --- | --- |
| Ref | Tar | HiRGC | Gain(%) | SCCG | Gain(%) | memRGC | Gain(%) | proposed |
| hg17 | hg18 | 10.8 | 79.6% | 10.8 | 79.6% | 2.4 | 8.3% | **2.2** |
|  | hg19 | 12.2 | 56.6% | 12.1 | 56.2% | 5.6 | 5.4% | **5.3** |
|  | hg38 | 20.7 | 37.2% | 20.4 | 36.3% | 13.8 | 5.8% | **13** |
|  | KO131 | 19.6 | 33.2% | 14.9 | 12.1% | 13.2 | 0.8% | **13.1** |
|  | KO224 | 18.1 | 35.9% | 13.3 | 12.8% | 11.7 | 0.9% | **11.6** |
|  | HuRef | 12.8 | 19.5% | 12.3 | 16.3% | 10.9 | 5.5% | **10.3** |
|  | YH | 13.5 | 43.7% | 8.7 | 12.6% | 7.7 | 1.3% | **7.6** |
| hg18 | hg17 | 9.9 | 84.8% | 9.9 | 84.8% | 1.6 | 6.3% | **1.5** |
|  | hg19 | 11.2 | 64.3% | 11.1 | 64.0% | 4.2 | 4.8% | **4** |
|  | hg38 | 19.9 | 39.2% | 19.7 | 38.6% | 12.9 | 6.2% | **12.1** |
|  | KO131 | 19.6 | 37.2% | 13.5 | 8.9% | **12.3** | 0.0% | **12.3** |
|  | KO224 | 17.1 | 36.3% | 12 | 9.2% | **10.8** | -0.9% | 10.9 |
|  | HuRef | 12.3 | 19.5% | 11.8 | 16.1% | 10.4 | 4.8% | **9.9** |
|  | YH | 12.5 | 44.8% | 7.6 | 9.2% | **6.8** | -1.5% | 6.9 |
| hg19 | hg17 | 10.2 | 62.7% | 10.1 | 62.4% | 4.1 | 7.3% | **3.8** |
|  | hg18 | 10.1 | 66.3% | 10 | 66.0% | 3.5 | 2.9% | **3.4** |
|  | hg38 | 19 | 50.0% | 18.9 | 49.7% | 10.2 | 6.9% | **9.5** |
|  | KO131 | 18.9 | 34.4% | 14.3 | 13.3% | 12.5 | 0.8% | **12.4** |
|  | KO224 | 17.4 | 36.8% | 12.7 | 13.4% | **11** | 0.0% | **11** |
|  | HuRef | 12.1 | 20.7% | 11.6 | 17.2% | 10.2 | 5.9% | **9.6** |
|  | YH | 12.8 | 45.3% | 8 | 12.5% | **7** | 0.0% | **7** |
| hg38 | hg17 | 11.6 | 43.1% | 11.5 | 42.6% | 7.9 | 16.5% | **6.6** |
|  | hg18 | 11.8 | 44.9% | 11.7 | 44.4% | 7.8 | 16.7% | **6.5** |
|  | hg19 | 12.1 | 62.0% | 12 | 61.7% | 5.8 | 20.7% | **4.6** |
|  | KO131 | 20.4 | 38.2% | 15.8 | 20.3% | 13.9 | 9.4% | **12.6** |
|  | KO224 | 18.8 | 40.4% | 14.2 | 21.1% | 12.4 | 9.7% | **11.2** |
|  | HuRef | 11.3 | 24.8% | 10.9 | 22.0% | 9.6 | 11.5% | **8.5** |
|  | YH | 14.3 | 49.7% | 9.5 | 24.2% | 8.4 | 14.3% | **7.2** |
| KO131 | hg17 | 20.1 | 14.9% | 18.8 | 9.0% | **16.9** | -1.2% | 17.1 |
|  | hg18 | 20.1 | 14.4% | 18.3 | 6.0% | **16.8** | -2.4% | 17.2 |
|  | hg19 | 21.4 | 14.5% | 20.4 | 10.3% | **18.1** | -1.1% | 18.3 |
|  | hg38 | 29.9 | 20.7% | 28.7 | 17.4% | 23.9 | 0.8% | **23.7** |
|  | KO224 | 9.3 | 52.7% | 4.7 | 6.4% | **4.3** | -2.3% | 4.4 |
|  | HuRef | 15.2 | 20.4% | 14.5 | 16.6% | 12.5 | 3.2% | **12.1** |
|  | YH | 14 | 45.0% | 8.4 | 8.3% | **7.5** | -2.7% | 7.7 |
| KO224 | hg17 | 20 | 15.0% | 18.7 | 9.1% | **16.8** | -1.2% | 17 |
|  | hg18 | 20 | 13.0% | 18.2 | 4.4% | **16.7** | -4.2% | 17.4 |
|  | hg19 | 21.3 | 14.6% | 20.3 | 10.3% | **18** | -1.1% | 18.2 |
|  | hg38 | 29.9 | 21.1% | 28.7 | 17.8% | 23.8 | 0.8% | **23.6** |
|  | KO131 | 10.7 | 56.1% | 6.1 | 23.0% | **4.6** | -2.2% | 4.7 |
|  | HuRef | 15.1 | 20.5% | 14.4 | 16.7% | 12.4 | 3.2% | **12** |
|  | YH | 13.8 | 44.9% | 8.2 | 7.3% | **7.3** | -4.1% | 7.6 |
| HuRef | hg17 | 36.1 | 18.3% | 34.9 | 15.5% | 32.2 | 8.4% | **29.5** |
|  | hg18 | 36.9 | 18.7% | 35.7 | 16.0% | 32.8 | 8.5% | **30** |
|  | hg19 | 37.9 | 19.0% | 36.6 | 16.1% | 33.7 | 8.9% | **30.7** |
|  | hg38 | 43.8 | 20.5% | 42.6 | 18.3% | 38 | 8.4% | **34.8** |
|  | KO131 | 38.6 | 29.0% | 33.7 | 18.7% | 30.2 | 9.3% | **27.4** |
|  | KO224 | 37 | 30.0% | 32.1 | 19.3% | 28.8 | 10.1% | **25.9** |
|  | YH | 33.2 | 32.8% | 28.1 | 20.6% | 25.3 | 11.9% | **22.3** |
| YH | hg17 | 18.5 | 14.1% | 17.4 | 8.6% | **15.8** | -0.6% | 15.9 |
|  | hg18 | 18.4 | 13.6% | 17.1 | 7.0% | **15.7** | -1.3% | 15.9 |
|  | hg19 | 19.8 | 14.1% | 18.9 | 10.1% | **17** | 0.0% | **17** |
|  | hg38 | 28.4 | 20.8% | 27.3 | 17.6% | 23 | 2.2% | **22.5** |
|  | KO131 | 18.5 | 37.3% | 13 | 10.8% | **11.5** | -0.9% | 11.6 |
|  | KO224 | 16.8 | 39.9% | 11.4 | 11.4% | **10** | -1.0% | 10.1 |
|  | HuRef | 14.7 | 21.1% | 14 | 17.1% | 12.1 | 4.1% | **11.6** |
| Total | | 1070.4 | 30.8% | 940.5 | 21.2% | 774.3 | 4.3% | **740.8** |

**Table 1:Compression results for 56 sets of experiments**

The compression time for all 56 pairs of genomes are shown in Table 2. Due to the significant difference in compression performance between HiRGC and SCCG and the proposed scheme, it is not reasonable to directly compare the compression speed gain with them. Therefore, in the following table, only the compression speed gain is compared with memRGC and the gain is calculated by formula (2).

$$\frac{\text{compression time of other algorithm-compression time of ours}}{\text{compression time of other algorithm}}\text{*}100\text{\% (2)}$$

| compression time (sec) | | | | | |  |
| --- | --- | --- | --- | --- | --- | --- |
| Ref | Tar | HiRGC | SCCG | memRGC | proposed | gain |
| hg17 | hg18 | 265.23 | 556.51 | 1221 | 549 | 55% |
|  | hg19 | 289.28 | 818.14 | 827 | 556 | 32.8% |
|  | hg38 | 287.59 | 616.12 | 666 | 554 | 16.8% |
|  | KO131 | 398.93 | 594.74 | 905 | 575 | 36.4% |
|  | KO224 | 396.25 | 639.49 | 862 | 576 | 33.2% |
|  | HuRef | 385.5 | 641.88 | 925 | 520 | 43.8% |
|  | YH | 368.18 | 584.11 | 853 | 596 | 30.1% |
| the whole genome |  | 2390.96 | 4450.99 | 6259 | 3926 | 37.3% |
| hg18 | hg17 | 261.04 | 667.87 | 1223 | 555 | 54.6% |
|  | hg19 | 269.84 | 813.5 | 810 | 554 | 31.6% |
|  | hg38 | 300.35 | 609.98 | 657 | 563 | 14.3% |
|  | KO131 | 381.63 | 468.1 | 859 | 577 | 32.8% |
|  | KO224 | 391.74 | 431.8 | 851 | 582 | 31.6% |
|  | HuRef | 388.72 | 635.32 | 939 | 530 | 43.6% |
|  | YH | 383.43 | 416.97 | 808 | 588 | 27.2% |
| the whole genome |  | 2376.75 | 4043.54 | 6147 | 3949 | 35.8% |
| hg19 | hg17 | 260.97 | 658.49 | 905 | 558 | 38.3% |
|  | hg18 | 264.05 | 639.3 | 910 | 557 | 38.8% |
|  | hg38 | 289.92 | 641.77 | 640 | 552 | 13.8% |
|  | KO131 | 385.95 | 649.88 | 903 | 583 | 35.4% |
|  | KO224 | 389.98 | 656.46 | 837 | 583 | 30.3% |
|  | HuRef | 386.02 | 617.92 | 920 | 532 | 42.2% |
|  | YH | 367.13 | 823.39 | 829 | 596 | 28.1% |
| the whole genome |  | 2344.02 | 4687.21 | 5944 | 3961 | 33.4% |
| hg38 | hg17 | 289.48 | 616.99 | 566 | 542 | 4.2% |
|  | hg18 | 280.54 | 645.82 | 566 | 551 | 2.7% |
|  | hg19 | 280.66 | 680.84 | 582 | 541 | 6.9% |
|  | KO131 | 406.51 | 768.21 | 986 | 578 | 41.4% |
|  | KO224 | 412.12 | 657.93 | 1005 | 630 | 37.3% |
|  | HuRef | 400.34 | 625.29 | 939 | 548 | 41.6% |
|  | YH | 390.47 | 804.44 | 965 | 646 | 33.1% |
| the whole genome |  | 2460.12 | 4799.52 | 5609 | 4036 | 28% |
| KO131 | hg17 | 392.66 | 560.05 | 878 | 535 | 39.1% |
|  | hg18 | 393.42 | 385.47 | 858 | 543 | 36.7% |
|  | hg19 | 379.56 | 676.35 | 893 | 541 | 39.4% |
|  | hg38 | 402.92 | 670.73 | 1089 | 573 | 47.4% |
|  | KO224 | 284.47 | 374.57 | 544 | 530 | 2.6% |
|  | HuRef | 403.28 | 644.93 | 992 | 537 | 45.9% |
|  | YH | 383.79 | 370.76 | 916 | 529 | 42.2% |
| the whole genome |  | 2640.1 | 3682.86 | 6170 | 3788 | 38.6% |
| KO224 | hg17 | 385.12 | 538.05 | 873 | 541 | 38% |
|  | hg18 | 385.27 | 441.18 | 855 | 536 | 37.3% |
|  | hg19 | 388.44 | 690.04 | 878 | 544 | 38% |
|  | hg38 | 399.23 | 660.78 | 1123 | 569 | 49.2% |
|  | KO131 | 283.72 | 380.91 | 544 | 524 | 3.7% |
|  | HuRef | 403.87 | 635.48 | 1024 | 537 | 47.6% |
|  | YH | 371.6 | 402.79 | 911 | 527 | 42.2% |
| the whole genome |  | 2617.25 | 3749.23 | 6208 | 3778 | 39.1% |
| HuRef | hg17 | 450.29 | 661.52 | 1198 | 567 | 52.7% |
|  | hg18 | 453.02 | 682.49 | 1207 | 575 | 52.4% |
|  | hg19 | 447.5 | 667.09 | 1184 | 577 | 51.3% |
|  | hg38 | 449.42 | 691.48 | 1308 | 607 | 53.6% |
|  | KO131 | 458.05 | 666.26 | 1249 | 613 | 50.9% |
|  | KO224 | 465.03 | 664.90 | 1220 | 610 | 50% |
|  | YH | 467.73 | 659.94 | 1250 | 625 | 50% |
| the whole genome |  | 3191.04 | 4693.68 | 8616 | 4174 | 51.6% |
| YH | hg17 | 357.51 | 525.59 | 847 | 537 | 36.6% |
|  | hg18 | 361.59 | 432.36 | 816 | 529 | 35.2% |
|  | hg19 | 361.03 | 882.41 | 891 | 540 | 39.4% |
|  | hg38 | 629.36 | 704.61 | 1032 | 566 | 45.2% |
|  | KO131 | 376.13 | 390.26 | 924 | 529 | 42.7% |
|  | KO224 | 372.75 | 381.56 | 900 | 534 | 40.7% |
|  | HuRef | 406.59 | 635.81 | 976 | 539 | 44.8% |
| the whole genome |  | 2864.96 | 3952.6 | 6386 | 3774 | 40.9% |

**Table 2. Compression Time for all 56 pairs of genomes**
